# Therapeutic potential of GLP-1 receptor agonists and SGLT2 inhibitors in diabetic neuropathy: a critical appraisal

**DOI:** 10.1186/s13098-026-02241-w

**Published:** 2026-07-30

**Authors:** Dan Ziegler, Peter Kempler, Cornelia Bala, Boris Mankovsky, Oliver Schnell, Vincenza Spallone, Alin Stirban, Solomon Tesfaye

**Affiliations:** 1 Institute for Clinical Diabetology, German Diabetes Center, Leibniz Center for Diabetes Research at Heinrich Heine University, Auf’m Hennekamp 65, 40225 Düsseldorf, Germany; 2 Department of Internal Medicine and Oncology, Semmelweis University, Budapest, Hungary; 3 Department of Diabetes and Nutrition Diseases, ‘‘Iuliu Hatieganu’’ University of Medicine and Pharmacy, Cluj-Napoca-Napoca, Romania; 4 Shupyk National Healthcare University of Ukraine, Kiev, Ukraine; 5 Forschergruppe Diabetes E.V., Helmholtz Munich (Helmholtz Zentrum München), Neuherberg (Munich), Neuherberg,, Germany; 6 Department of Systems Medicine, Endocrinology Section, University of Rome Tor Vergata, Rome, Italy; 7 Asklepios Klinik Birkenwerder, Heinrich Heine University, Birkenwerder, Düsseldorf, Germany; 8 Diabetes Research Unit, Sheffield Teaching Hospitals NHS Foundation Trust, Sheffield, UK

## Abstract

The management of type 2 diabetes (T2D) has recently witnessed a paradigm shift extending beyond blood glucose regulation towards cardiovascular and renal health targeted by cardiovascular outcome trials (CVOTs) of glucagon-like peptide-1 receptor agonists (GLP-1RAs) and sodium-glucose cotransporter-2 inhibitors (SGLT2i). Preclinical studies suggest a potential role for GLP-1RAs and SGLT2i in the treatment of diabetic neuropathy and neuropathic pain. However, to date largely only small-size phase II shorter-term randomized controlled trials (RCTs) of heterogeneous designs and quality have been performed, without clear evidence of favorable effect of both GLP-1RAs and SGLT2i on clinical and neurophysiological neuropathic outcomes. Moreover, responses to GLP1-RA and SGLT2i treatments in real‑world clinical practice appear heterogeneous, with relatively high rates of non-responders and discontinuation. Several possible risks associated with GLP-1RAs and SGLT2i treatment in real-world practice have been identified requiring increased attention by physicians, their professional societies, and patients alike. Unfortunately, the opportunity has been missed to use simple tools for the detection and monitoring of diabetic sensorimotor polyneuropathy (DSPN) and cardiovascular autonomic neuropathy (CAN) in the multiple published large-scale pivotal CVOTs. Diabetic neuropathy is linked to considerable patient burden and mortality, and there remains an unmet need for the disease modifying effects of novel pharmacotherapies derived from the pathogenetic concepts of diabetic neuropathy. In the future, when designing and conducting RCTs more emphasis should be placed on considering neuropathy as a serious and potentially life-threatening complication.

## Introduction

Over the past decade, the management of type 2 diabetes (T2D) has been transformed by two classes of glucose-lowering drugs: glucagon-like peptide-1 receptor agonists (GLP-1RAs) and sodium-glucose cotransporter-2 inhibitors (SGLT2i), both of which offer therapeutic advantages extending beyond blood glucose regulation. Incretin peptides, specifically glucagon-like peptide-1 (GLP-1) and glucose-dependent insulinotropic polypeptide (GIP), are essential metabolic regulators now utilized as potent pharmacotherapies. While GLP-1RAs are established treatments for T2D and obesity, recent innovations have introduced long-acting single-molecule dual- and multi-agonists targeting GLP-1, GIP, and glucagon receptors to improve clinical outcomes. Agents such as semaglutide and tirzepatide have recently gained unprecedented global attention from both the medical community and the public. Beyond glycemic and weight control, these therapies demonstrate significant benefits for cardiovascular and renal health [1–4].

The therapeutic rationale for SGLT2 inhibition in diabetes is rooted in the transporter’s role in sustaining hyperglycemia. By blocking SGLT2, these inhibitors trigger a consistent urinary glucose excretion of 40–80 g/day, typically reducing HbA1c levels in people with T2D by 0.5–0.7% [5]. Because the volume of glucose filtered and subsequently excreted depends on blood glucose levels and glomerular filtration rate (GFR), the efficacy of SGLT2i scales with these factors. However, this same glucosuric mechanism predisposes patients to an elevated risk of genitourinary infections, the primary side effect of the class [5]. Similar to GLP-1RAs, the clinical application of SGLT2i has expanded rapidly over the past decade.

Diabetic neuropathies rank among the most frequent complications of diabetes, with diabetic sensorimotor polyneuropathy (DSPN) being the most frequent manifestation affecting around one third of people with diabetes and contributing to an increased risk of mortality and considerable morbidity, it remains inadequately diagnosed and treated [6–9]. Notably, recent evidence suggests that among metabolic diseases neuropathy is not only present in diabetes, but also in prediabetes [10], obesity [11], and the metabolic syndrome [12]. Management of DSPN includes three cornerstones: 1.) lifestyle modification, optimal diabetes treatment aimed at (near-)normoglycemia, and multifactorial cardiovascular risk intervention, 2.) pathogenetic pharmacotherapy, and 3.) symptomatic treatment of neuropathic pain including analgesic pharmacotherapy and non-pharmacological options [8, 13]. Although intensive glucose-lowering strategies have been shown to partially prevent the development or slow the progression of DSPN including neuropathic pain in individuals with type 1 diabetes (T1D) [14], there is no consistent evidence for those with T2D [15]. Nonetheless, there is general agreement that glucose control should be optimized in people both with T1D and T2D [6, 8, 16].

Major and difficult-to-treat sequels of DSPN include neuropathic pain and foot ulcers leading to markedly reduced quality of life and increased risk of mortality [6, 8, 9, 17]. Symptomatic analgesic pharmacotherapy only relieves the pain without targeting the underlying neuropathy [8, 10], and has limited efficacy with 54% response at the best with combination therapy [18]. Unfortunately, efficacy of analgesic agents is sometimes restricted by dose limiting adverse events [6, 8, 9, 16]. Despite some available agents such as α-lipoic acid, which has robust evidence in DSPN [16, 19, 20], there remains an unmet need for novel pharmacotherapies derived from the pathogenetic mechanisms leading to diabetic neuropathy. In this review we discuss the potential of GLP-1 RAs and SGLT2i as disease modifying pharmacotherapies for diabetic neuropathies with particular emphasis on the mechanistic concepts and the evidence for efficacy and safety from RCTs. We also discuss the more general aspects of GLP-1 RAs and SGLT2i use in real-world practice, some of which are not directly related to DSPN including the risks as well as the response and discontinuation rates which have to be weighed against the available pathogenetic treatments such as α-lipoic acid that show an excellent safety profile without major side effects [19] and, hence, are suitable for long-term treatment.

## Metabolic, cardiovascular, and renal benefits

Originally designed for the therapy of T2D, GLP-1RAs have revolutionized treatment not only by providing superior glycemic control, promoting weight loss, and maintaining a low hypoglycemia risk, but also, beyond these metabolic benefits, by lowering the incidence of major adverse cardiovascular events (MACE) and heart failure hospitalizations [2, 3, 21]. Their renal protective effects include reducing albuminuria and slowing GFR decline, thereby delaying kidney failure [21] (Table 1). Liraglutide, semaglutide, and the dual GIP/GLP-1RA tirzepatide are now approved for obesity, showing efficacy in treating comorbidities such as steatotic liver disease, obstructive sleep apnea, and osteoarthritis. Future directions involve oral small-molecule formulations and multi-receptor (dual or triple) agonists to enhance weight loss and target neurological or addictive disorders [1, 2].

**Table 1:** Benefits of GLP-1RAsand SGLT2 inhibitors in T2D, showing comparative effect estimates versus standard treatments (adapted from ref. 21)

**Outcomes**	**GLP-1 RAs** **OR (95% CI)**	**SGLT2 inhibitors** **OR (95% CI)**
All-cause mortality	**0.87 (0.82 to 0.92)**	**0.88 (0.83 to 0.94)**
Non-fatal myocardial infarction	**0.91 (0.85 to 0.98)**	**0.90 (0.82 to 0.98)**
Non-fatal stroke	**0.87 (0.79 to 0.96)**	***0.99 (0.88 to 1.11)***
3-P MACE	**0.85 (0.80 to 0.91)**	**0.89 (0.83 to 0.95)**
Hospitalization for heart failure	*0.91 (0.83 to 0.99)*	**0.66 (0.60 to 0.72)**
Kidney disease progression	*0.84 (0.76 to 0.93)*	**0.61 (0.55 to 0.69)**

High to moderate certainty of evidence: Bold: Among the most effective; Italic: Among the intermediate effective. Low to very low certainty of evidence: Bold italic: Possibly among the intermediate effective. 3-P MACE: 3-point major adverse cardiovascular events: death from a cardiovascular cause, non-fatal stroke, or non-fatal myocardial infarction

The American Diabetes Association (ADA) recommends a GLP-1RA or dual GIP/GLP-1RA (tirzepatide) particularly for adults with T2D (irrespective of HbA1c) and established or high risk of atherosclerotic CVD, those with T2D, obesity and symptomatic heart failure with preserved ejection fraction (HFpEF), and for those with T2D and chronic kidney disease to reduce symptoms and events. Furthermore, GLP-1 RAs are preferred for glycemic management in adults with T2D and metabolic dysfunction-associated steatohepatitis (MASH), especially those at high risk for liver fibrosis. The ADA also supports the use of GLP-1 RAs in adults with T1D who have a BMI > 30 (or > 27.5 for Asian Americans) to assist with weight and metabolic health, provided there is close monitoring for ketosis [22].

Similar to GLP-1RAs, the role of SGLT2i has expanded to broader cardio-renal protection based on evidence from individuals with CKD and heart failure. The benefits of SGLTi on cardiovascular and renal outcomes in people with T2D are summarized in Table 1 [21]. Current clinical practice guidelines recommend the use of SGLT2i in individuals with CKD (eGFR 20 mL/min/1.73 m^2^) who also have diabetes, heart failure, or significant albuminuria (urinary albumin to creatinine ratio: UACR > 200 mg/g) [23]. However, in a recent meta-analysis SGLT2i were found to lower the risk of CKD progression regardless of baseline eGFR or UACR, including in patients with stage 4 CKD or minimal albuminuria, supporting their routine use to improve kidney outcomes across the full spectrum of kidney function among patients with T2D, CKD, or heart failure [24]. Further analyses in participants stratified by diabetes status and UACR (≥ 200 mg/g or < 200 mg/g) demonstrated that clear benefits of SGLT2i on kidney, hospitalization, and mortality outcomes were achieved irrespective of diabetes status and UACR levels [25]. While the cardiovascular and renal benefits of SGLT2i are undisputed, they have to be weighed against drug specific adverse events such as genital infections (OR [95% CI]: 3.29 [2.88–3.77]), ketoacidosis (2.08 [1.45–2.99]), and possibly amputations (1.27 [1.01–1.61]) [21].

Indirect comparisons of GLP1RAs with SGLT2i show distinct profiles for the two drug classes. The mean differences in weight and HbA1c reduction were -1.94 (-2.14 to -1.74) kg and -0.59 (-0.64 to -0.55) % for SGLT2 inhibitors, -4.44 (-5.02 to -3.86) kg and -1.36 (-1.51 to -1.21) % for s.c. semaglutide, -2.89 (-3.51 to -2.26) kg and -1.15 (-1.31 to -0.99) for oral semaglutide, and -8.63 (-9.34 to -7.93) kg and -1.78 (-1.96 to -1.59) % for tirzepatide [21], respectively. While heart failure hospitalization and composite renal outcomes were more favorably influenced by SGLT2i than GLP-1RAs (Table 1) [21, 26], the latter were associated with a nominally greater MACE reduction (HR 1.09, 95% CI 1.00–1.18) than SGLT2i in a recent meta-analysis [26].

## Rationale for pathogenetic pharmacotherapy of diabetic neuropathy

### GLP-1 receptor agonists

Although incretin receptors are widely distributed throughout the body including the hypothalamus, their role in the peripheral nervous system (PNS) remains largely understudied. This apparent negligence is surprising, given that the PNS drives key metabolic processes in the liver, gut, and fat tissues which are supplied by nerves that express these very receptors. Since a critical component of metabolic disease involves the dysregulation of neural pathways governing energy homeostasis, both centrally and peripherally, the activity of incretins and their pharmacological analogs across both branches of the nervous system is likely fundamental to their therapeutic efficacy. Nonetheless, research has predominantly focused on central nervous system (CNS) rather than PNS related mechanisms [27]. However, there is substantial evidence suggesting that GLP-1 receptors are expressed in vagal afferents, a key component of the PNS, being necessary for glucose homeostasis and satiety regulation. GLP-1R was also found expressed in immortalized rat neurons, Schwann cells, innervation in the hepatic portal bed, enteric nervous system, sciatic nerve, and dorsal root ganglia (DRGs) [27]. GIP receptor expression and function in the PNS are less well characterized, but GIP receptor expression has been found in the DRG and sciatic nerve, indicating that GIP receptor activity is essential for peripheral neuron health [28]. Supporting these findings, recombinant GIP treatment following sciatic nerve transection improved nerve repair and motor function, while promoting axonal extension and Schwann cell proliferation [29].

A bidirectional interaction exists between the PNS and incretin hormones, whereby the PNS both responds to and regulates incretin secretion. Muscarinic receptor expression on human and rodent enteroendocrine L cells allows for the stimulation of GLP-1 in vitro upon activation. While enteric neuronal activity promotes this cholinergic-mediated secretion, norepinephrine exerts an inhibitory effect on GLP-1 release, as demonstrated in porcine ileal preparations [30]. Preclinical research supports the therapeutic potential of incretin-based drugs for treating peripheral nerve diseases and lesions. In various rat models, exenatide treatment improved nerve morphology following infraorbital nerve injury, accelerated recovery after sciatic nerve transection and crush injuries, and preserved nerve function in streptozotocin-induced diabetic neuropathy [27, 31–33].

Furthermore, emerging preclinical evidence highlights the therapeutic potential of GLP-1RAs in alleviating neuropathic pain. Their favorable effects on pain are mediated through the modulation of microglial activity, suppression of inflammatory cytokine production, and stimulation of β-endorphin release [34]. By inhibiting key inflammatory signaling pathways, GLP-1RAs demonstrated significant analgesic efficacy, particularly in chronic pain states. Of note, unlike some traditional analgesics, GLP-1RAs achieve these effects without the development of pharmacological tolerance, representing a major advantage for long-term pain management [34].

These findings are supported by data showing that in participants with overweight or obesity from the STEP 1 and STEP 2 RCTs, proteins downregulated with neuropathic pain in the real-world proteomic profiles in the deCODE study were upregulated by semaglutide, while proteins upregulated with neuropathic pain in the deCODE study were downregulated by semaglutide, contributing to the concept of GLP-1RAs actions beyond weight loss and glucose lowering [35]. Given that oxidative stress has been identified as the initial event in the pathogenesis of diabetic microvascular complications including neuropathy [36], it is noteworthy that treatment with both GLP-1RAs and SGLT2i in individuals with T2D was shown to improve various biomarkers of oxidative stress as reported in a recent meta-analysis of RCTs [37].

Collectively, these data suggest the further exploration of the physiological role of incretins in the PNS and their potential as disease modifying pharmacotherapies in diabetic neuropathy and neuropathic pain in the clinical setting.

### SGLT2 inhibitors

Experimental studies suggest neuroprotective effects of SGLT2 inhibitors. In obese mice with T2D, empagliflozin increased cerebral levels of brain-derived neurotrophic factor (BDNF) by alleviating neuronal oxidative stress [38]. BDNF critically governs the development of brain circuits, synaptic plasticity, neuroregeneration, and neuroprotection, playing a pivotal role in synaptic transmission [39]. In mouse models of T1D, empagliflozin improved electrophysiological parameters, tactile hypersensitivity, and IENFD [40, 41], while dapagliflozin improved locomotor behavior and nociceptive sensitivity, and nerve conduction attributes. Histological analyses showed decreased hydroxynonenal (HNE) immunoreactivity, suggesting attenuation of oxidative stress, reduced perineural fibrogenesis, and increased IENFD [42]. Thus, there is evidence suggestin that SGLT2i offer robust neuroprotection against diabetes-induced neurodegeneration and neuronal dysfunction. These protective effects are primarily driven by enhancing mitochondrial function, while simultaneously suppressing oxidative stress, inflammation, and cellular death pathways in affected neurons [43].

On the other hand, by lowering insulin and stimulating pancreatic alpha cells, SGLT2i increase glucagon secretion. This high glucagon-to-insulin ratio acts as a powerful fasting signal to adipose tissue, accelerating lipolysis and elevating circulating free fatty acids (FFAs) and triglycerides (TGs) [44, 45]. While these lipids provide the raw materials for ketone production, which acts as an efficient fuel source for the heart and kidneys (Ferrannini), they may present a distinct challenge for peripheral nerves. Elevated FFAs and TGs can flood Schwann cells and axons, causing mitochondrial overload, oxidative stress, and the accumulation of toxic lipid metabolites like ceramides [46, 47]. This localized lipotoxicity can damage the myelin sheath and impair axonal function, potentially aggravating diabetic neuropathy [46]. This dual nature points to a metabolic paradox where systemic benefits may co-exist with localized peripheral nerve risks.

With respect to autonomic modulation, evidence has emerged to suggest a crosstalk between the sympathetic nervous system (SNS) and SGLT2 regulation. SNS activation appears to upregulate SGLT2, whereas SGLT2 inhibitors exert sympathoinhibitory effects. Beyond regulating cortical vasoconstriction, efferent sympathetic fibers promote sodium reabsorption in the renal proximal tubular epithelial cells which are also targeted by SGLT2is [48]. Norepinephrine was shown to increase SGLT2 expression in human proximal tubular cells [49], whereas chemical denervation with 6-hydroxydopamine (6-OHDA) reduced renal SGLT2 expression in neurogenic hypertensive mice [50]. Likewise, renal sympathetic denervation in obese diabetic OLETF rats suppressed SGLT2 overexpression [51]. SGLT2i such as dapagliflozin have been shown to inhibit tyrosine hydroxylase and norepinephrine expression in the kidneys and hearts of high-fat-diet-fed mice [49], while empagliflozin improved renal sympathetic baroreflex sensitivity (BRS) in diabetic rabbits [52]. These interactions may be potentially relevant in conditions characterized by sympathetic overactivity, such as T2D, obesity, hypertension, and obstructive sleep apnea [48].

Elevated sympathetic activity is a key driver in the development and persistence of cardiac arrhythmias and heart failure. Dapagliflozin treatment was found to normalize sympathetic nerve activity in pig models of heart failure with preserved ejection fraction [53]. This sympatholytic effect is likely driven by SGLT2i’s properties to lower hyperglycemia and tyrosine hydroxylase expression, reduce circulating leptin, and stimulate ketone body production, which inhibits norepinephrine release via FFAR3/GPR41 receptor blockade [54]. Another mechanism may involve reducing stimuli in the intrarenal microenvironment, such as hypoxia and intraglomerular pressure, which lowers afferent renal nerve activity to the central nervous system and hypothalamus, thus decreasing sympathetic outflow to the heart and kidneys [48, 55].

Collectively, the experimental evidence provides a rationale to investigate the potential of SGLT2 inhibitors as disease-modifying agents in patients with DSPN and autonomic neuropathy.

## Evidence from controlled clinical trials

### Diabetes/prediabetes with or without neuropathy

Four placebo controlled RCTs (3 of which used liraglutide) conducted over 2.8–7 months assessed the effects of GLP-1RAs on measures of neuropathy in participants with diabetes or prediabetes with or without DSPN [56–60] (Table 2). In these studies none of the primary and secondary endpoints of DSPN and CAN showed improvement in favor of GLP-1RA treatment, but two studies reported an increase in heart rate after liraglutide treatment [59, 60], while in one study several HRV measures worsened after 2.8 months of liraglutide administration [60]. Only the post-hoc analysis by Wegeberg et al. [58] reported an accelerated colonic transit time and improved motility index in response to liraglutide treatment, but postprandial fullness worsened, while small bowel transit time and gastric emptying remained unchanged. In participants with prediabetes, overweight/obesity, and schizophrenia who received treatment with second-generation antipsychotics no effect on early or definite CAN was observed after 7 months of treatment with semaglutide [56] (Table 2).

**Table 2:** Randomized double-blind placebo controlled clinical trials that assessed the effects of GLP-1RAs on measures of peripheral and autonomic function in individuals with diabetes or prediabetes

**Author**	**Ganeshalingam et al., 2026 **[56]	**Brock et al., 2019 **[57]**, Wegeberg et al., 2020 **[58]	**Hansen et al., 2019 **[59]	**Kumarathurai et al., 2017 **[60]*
Diabetes type	Prediabetes	1	1	2 (newly diagnosed)
Study population	Prediabetes (84% with CAN) + schizophrenia	Confirmed DSPN	Overweight/obese T1D	T2D + CAD
n (active/control)	74/67	19/20	50/49	19/20
Treatment/control	Semaglutide/placebo	Liraglutide/placebo	Liraglutide/placebo	Liraglutide/placebo
Treatment duration (months)	7	6	5.6	2.8
Primary outcome(s)	· Early/definite CAN ↔	· SEP: precortical N20 latency ↔	· E/I, 30:15, Valsalva ratios↔ · SDNN, RMSSD↔ · HF, LF, TP, LF/HF↔ · HR ↓ (increase)	24 h Holter monitoring: · SDNN ↓ · SDNN index ↓ · SDANN ↓ · RMSSD ↓ · LF, HF, TP ↓ · LF/HF↔ · HR ↓ (increase) · HR nighttime ↓ (increase) · HR daytime↔
Secondary outcomes		· Other SEP latencies/amplitudes ↔ · Median, tibial, radial MNCV ↔ · Sural SNCV and SNAP ↔ · Ulnar, radial, tibial CMAP ↔ · HR, SDNN, SDANN, RMSSD, VLF, LF, HF, LF/HF ↔ · Colonic transit time ↑ · Motility index ↑ · Postprandial fullness ↓ · Gastric emptying ↔ · Small-bowel transit time ↔		

↑Improvement after treatment vs control, ↓ worsening after treatment vs control, ↔ no difference between groups; *crossover.

CAD: coronary artery disease; CAN: cardiovascular autonomic neuropathy; MNCV: motor nerve conduction velocity; SNCV: sensory nerve conduction velocity; SNAP: sensory nerve action potential; CMAP: compound muscle action potential; LF: low frequency power spectrum; HF: high frequency power spectrum; HR: heart rate; RMSSD: root mean squared successive difference; SDNN: standard deviation of normal-to-normal intervals; SDANN: standard deviation of the average normal-to-normal intervals; DSPN: diabetic sensorimotor polyneuropathy; SEP: somatosensory evoked potentials; TP: total power spectrum

Three open-label RCTs conducted over 4.2–18 months investigated the effects of GLP-1RAs on measures of DSPN and/or CAN in participants with T2D with or without DSPN [61–63] (Table 3). Two trials used insulin treatment as the only comparator [61, 63], while one trial used a combination of a GLP-1RA and pioglitazone [61]. Similar to the placebo controlled RCTs, none of the primary and secondary endpoints of DSPN and CAN showed improvement in favor of GLP-1RA treatment, but in the study by Ponirakis et al. [61] vibration perception threshold (VPT) worsened in the GLP-1RA + pioglitazone group as compared to the control group, while Nyström et al. [62] reported an increase in daytime heart rate (Table 3).

**Table 3:** Randomized open-label controlled clinical trials that assessed the effects of GLP-1RAs on measures of peripheral and autonomic function in individuals with type 2 diabetes

**Author**	**Ponirakis et al., 2020 **[61]	**Nyström et al., 2019 **[62]	**Jaiswal et al., 2015 **[63]
Study population	T2D	T2D + EF ≤ 50%/diastolic dysfunction	T2D + confirmed mild to moderate DSPN
n (active/control)	21/17	33/29	22/24
Treatment/control	Exenatide + pioglitazone/glargine + aspart*	Liraglutide/glimepiride	Exenatide/glargine
Treatment duration (months)	12	4.2	18
Primary outcome(s)	· CNFL ↔ · CNFD ↔ · CNBD ↔	24 h Holter monitoring: · SDNN, SDNN index, SDANN, RMSSD, pNN50↔ · VLF, LF, HF, TP↔ · LF/HF↔ · HR↔ · HR daytime ↓ (increase) · HR nighttime↔	· Composite score: confirmed clinical DSPN ↔
Secondary outcomes	· VPT ↓ · DN4 ↔ · ESC feet ↔		· Median, peroneal MNCV, CMAP, FRL ↔ · Median, sural SNCV, SNAP↔ · Symptoms, signs ↔ · HR, SDNN, RMSSD, LF/HF, E/I, Valsalva ↔ · NeuroQoL ↔ · IENFD [12 months] ↔

↑Improvement after treatment vs control, ↓worsening after treatment vs control, ↔ no difference between groups; *combination therapy.

CNFL: corneal nerve fiber length; CNFD: corneal nerve fiber density; CNBD: corneal nerve branch density; MNCV: motor nerve conduction velocity; SNCV: sensory nerve conduction velocity; SNAP: sensory nerve action potential; CMAP: compound muscle action potential; EF: ejection fraction; E/I: expiration/inspiration ratio; FRL: F wave response latency; QoL: quality of life; VPT: vibration perception threshold; VLF: very low frequency power spectrum; LF: low frequency power spectrum; HF: high frequency power spectrum; HR: heart rate; RMSSD: root mean squared successive difference; ESC: electrochemical skin conductance; DN4: Douleur Neuropathique en 4 Questions questionnaire; SDNN: standard deviation of normal-to-normal intervals; SDANN: standard deviation of the average normal-to-normal intervals; DSPN: diabetic sensorimotor polyneuropathy; n.a.: not applicable; TP: total power spectrum; IENFD: intraepidermal nerve fibre density; NeuroQoL: Neuropathy- and foot ulcer-specific quality of life instrument

Overall, these rather heterogeneous small trials do not provide any evidence for a favorable effect of GLP-1RAs on measures of DSPN and CAN, while the putative positive large bowel effect would require confirmation. Likewise, a meta-analysis could not identify any benefit of GLP-1RAs on measures of DSPN [64], while the outcome of another meta-analysis was biased by observational studies [65]. A placebo controlled, 32-week RCT using CagriSema (once weekly s.c.), a combination of semaglutide and the long-acting amylin analogue cagrilintide, in overweight or obese individuals with T2D and painful DSPN is currently underway (https://clinicaltrials.gov/study/NCT06797869).

Four placebo controlled RCTs conducted over 3–29.4 months investigated the effects of SGLT2i on measures of DSPN and/or CAN in participants with T2D with or without DSPN [66–69] (Table 4). One trial using measures of autonomic function did not show any favorable effect of empagliflozin on multiple primary and secondary time and frequency domain indices of heart rate variability (HRV) [69]. The other two trials conducted in participants with DSPN suggested some benefit of empagliflozin on nerve conduction studies and neuropathic symptoms, but not on neurological examination scores [66, 67]. Moreover, Vafaeinasab et al. [66] did not specify a primary endpoint, while El-Haggar et al. [67] used multiple primary endpoints without providing an appropriate sample size calculation required to detect any clinically meaningful minimal difference between the active and control arm. Although these studies were somewhat larger than those using GLP-1RAs, the samples per arm were still relatively small, and the maximum study duration was only 5.5 months (Table 4).

**Table 4:** Randomized placebo controlled clinical trials assessing the effects of SGLT2 inhibitors on measures of peripheral and autonomic function in individuals with T2D

Author	Vafaeinasab et al., 2025 [66]	El-Haggar et al., 2024 [67]	Liao et al., 2022 [68]	Shimizu et al., 2020 [69]
RCT design	Double blind	Single blind	Double blind	Double blind
Study population	DSPN	Confirmed DSPN	CKD (49% with DSPN)	AMI
n (active/placebo)	25/25	25/25	2202/2199	46/50
Treatment/placebo	Empagliflozin/placebo	Empagliflozin/placebo	Canagliflozin/placebo	Empagliflozin/placebo
Treatment duration (months)	4.7	3	29.4	5.5
Primary outcome(s)	· MNSI-Q ↑ · MNSI-E ↔ · Peroneal, radial MNCV, latency ↑ · Peroneal, radial CMAP ↔ · Sural SNCV, latency ↑ · Sural SNAP ↔	· Peroneal, tibial MNCV, CMAP, latency ↑ · Sural SNCV, SNAP, latency ↑	· All neuropathy events ↔	· SDNN, SDANN, RMSSD, pNN50 ↔ · LF, HF, LF/HF ↔
Secondary outcomes		· BPI-SF ↑ · DNS ↔	Subcategory events: · DSPN ↔ · DN ↔ · N-AN ↔	· MIBG scintigraphy ↔ · HRT ↔ · LP ↔ · TWA ↔

↑ Improvement after treatment vs control, ↔ no difference between groups.

MNCV: motor nerve conduction velocity; SNCV: sensory nerve conduction velocity; SNAP: sensory nerve action potential; CMAP: compound muscle action potential; LF: low frequency power spectrum; HF: high frequency power spectrum; RMSSD: root mean squared successive difference; SDNN: standard deviation of normal-to-normal intervals; SDANN: standard deviation of the average normal-to-normal intervals; DSPN: diabetic sensorimotor polyneuropathy; pNN50: percentage of successive normal-to-normal intervals > 50 ms; BPI-SF: Brief Pain Inventory Short-Form item score; DNS: Diabetic Neuropathy Score; CKD: chronic kidney disease; MNSI-E/Q: Michigan Neuropathy Screening Instrument – Examination/Questionnaire; DN: diabetic neuropathy; N-AN: non-autonomic neuropathy; TWA: T-wave alternans, LP: late potentials, HRT: heart rate turbulence; AMI: acute myocardial infarction; DN4: Douleur Neuropathique en 4 Questions questionnaire; MIBG: meta-iodobenzylguanidine; RCT: randomized controlled trial

In a post-hoc analysis of the CREDENCE trial, neuropathy events were defined as any reported adverse event consistent with DSPN, diabetic neuropathy, and non-autonomic neuropathy events. The incidence of overall neuropathy events was similar in people randomized to canagliflozin and placebo (HR 1.04, 95% CI 0.89–1.21). Moreover, canagliflozin had no effect on DSPN, diabetic neuropathy, or non-autonomic neuropathy [68] (Table 4).

Five open-label RCTs conducted over 2.8–6 months investigated the effects of SGLT2i on measures of DSPN and/or CAN in participants with T1D or T2D with or without DSPN [70–74] (Table 5). The three RCTs focusing on CAN did not show any favorable effects of empagliflozin and dapagliflozin on multiple time and frequency domain HRV measures and cardiovascular autonomic reflex tests [71–73]. Only two trials showed some minor benefit on DSPN endpoints [70, 74]. However, Mehta et al. [74] used a combination of empagliflozin and vitamin D which was associated with an improvement of neuropathic pain and other symptoms as well as improved touch sensation, but empagliflozin monotherapy was not better than standard of care. Since all patients had low vitamin D levels at baseline, these results are difficult to interpret but do not suggest benefit for empagliflozin monotherapy.. Adhikari et al. [70] used multiple primary endpoints without providing an appropriate sample size calculation. They reported improvement in corneal nerve morphology and neuropathic symptoms with dapagliflozin, but no effect of this SGLT2i on IENFD and VPT (Table 5).

**Table 5:** Randomized controlled clinical trials that assessed the effects of SGLT2 inhibitors on measures of peripheral and autonomic function in individuals with T2D

**Author**	**Adhikari et al., 2025 **[70]	**Motoki et al., 2025 **[71]	**Laursen et al., 2023 **[72]	**Ang et al., 2021 **[73]	**Mehta et al., 2021 **[74]
RCT design	Open-label	Open-label	Open-label CO	Single blind CO	Open-label*
Study population	Symptomatic DSPN	T2D	T1D	T2D	Painful DSPN + low vitamin D
n (active/control)	22/18	55/54	26/26/26/26	19/26	50/50/50
Treatment/control	Dapagliflozin/SOC	Empagliflozin/ sitagliptin	Empagliflozin/ linagliptin/ telmisartan/ baricitinib	Dapagliflozin/ glimepiride	Empagliflozin + vitamin D/empagliflozin/SOC
Treatment duration (months)	6	5.5	1	2.8	6
Primary outcome(s)	· CNFD ↑ · CNBD ↑ · CNFL ↔ · IENFD ↔	· LF/HF ratio ↔	· SDNN ↔	· LF/HF ratio ↔	Empagliflozin + vitamin D: · DN4 ↑ · NPSI ↑ · ITTT ↑ Empagliflozin mono: · DN4 ↔ · NPSI ↔ · ITTT ↔
Secondary outcomes	· MNSI-E ↑ · VPT ↔	· SDNN, AVNN, RMSSD, pNN50 ↔ · LF, HF ↔	· LF, HF ↔ · E/I, Valsalva ratios ↔	· SDNN, RMSSD ↔ · E/I, 30:15, Valsalva ratios ↔	

↑ Improvement after treatment vs control, ↔ no difference between groups; *combination therapy.

CNFL: corneal nerve fiber length; CNFD: corneal nerve fiber density; CNBD: corneal nerve branch density; IENFD: intraepidermal nerve fiber density; VPT: vibration perception threshold; LF: low frequency power spectrum; HF: high frequency power spectrum; RMSSD: root mean squared successive difference; SDNN: standard deviation of normal-to-normal intervals; AVNN: average of normal-to-normal intervals; DSPN: diabetic sensorimotor polyneuropathy; pNN50: percentage of successive normal-to-normal intervals > 50 ms; MNSI-E: Michigan Neuropathy Screening Instrument – Examination; ITTT: Ipswich Touch the Toes Test; AMI: acute myocardial infarction; DN4: Douleur Neuropathique en 4 Questions questionnaire; CO: crossover; NPSI: Neuropathic Pain Symptom Inventory; RCT: randomized controlled trial

A living systematic review and network meta-analysis using frequentist random effects and GRADE (grading of recommendations, assessment, development and evaluation) approaches analyzed 13 drug classes (63 drugs) including GLP-1RAs, tirzepatide, and SGLT2i for 26 outcomes in 493,168 participants from 869 RCTs of at least 24 weeks comparing one or more medications with standard treatment, placebo, or each other [21]. Updates of this analysis are planned at least two times a year. The neuropathy outcome was defined as the number of patients who suffered from diabetic neuropathy, neuropathic pain, or peripheral neuropathy (Table 6). The certainty of evidence in this analysis was very low, except for the comparison of GLP-1RAs with DPP-4 inhibitors which was low and the comparison of GLP-1RAs with standard treatment and sulfonylurea which was moderate. The only significant benefit in neuropathy outcome was found for GLP-1RAs compared to sulfonylureas. In contrast, all other comparisons of GLP-1RAs and all comparisons of both tirzepatide and SGLT2i with other treatments did not show any favorable effects (Table 6) [21]. Likewise, a meta-analysis could not identify any benefit of SGLT2i on measures of CAN [75], while the results of another meta-analysis were biased by observational studies [76].

**Table 6:** Network meta-analysis: effects of glucose lowering drugs on neuropathy (adapted from ref. 21)

	**GLP-1 RAs** **OR (95% CI)**	**Tirzepatide** **OR (95% CI)**	**SGLT2 inhibitors** **OR (95% CI)**
Basal insulin	*1.06 (0.91, 1.23)*	*0.78 (0.09, 6.83)*	*1.05 (0.82, 1.35)*
Basal-bolus insulin	*0.48 (0.10, 2.27)*	*0.36 (0.02, 5.09)*	*0.48 (0.10, 2.29)*
Bolus insulin	*2.75 (0.72, 10.49)*	*2.03 (0.37, 11.13)*	*2.74 (0.71, 10.60)*
DPP-4 inhibitors	***1.09 (0.97, 1.23)***	*0.80 (0.09, 7.00)*	*1.08 (0.87, 1.35)*
Finerenone	*1.19 (0.59, 2.37)*	*0.87 (0.09, 8.48)*	*1.18 (0.58, 2.40)*
Metformin	*0.96 (0.49, 1.91)*	*0.77 (0.08, 7.41)*	*1.03 (0.51, 2.09)*
Standard treatments	***1.01 (0.92, 1.11)***	*0.73 (0.08, 6.25)*	*1.02 (0.84, 1.22)*
Sulfonylureas	***0.83 (0.71, 0.96)***	*0.89 (0.10, 7.79)*	*0.83 (0.65, 1.07)*
Thiazolidinediones	*0.83 (0.66, 1.06)*	*0.88 (0.10, 7.79)*	*0.84 (0.63, 1.12)*
GLP-1 receptor agonists	-	-	*0.99 (0.81, 1.22)*
Tirzepatide	*0.74 (0.08, 6.42)*	-	-
SGLT2 inhibitors	-	*0.74 (0.08, 6.52)*	-

GRADE guidelines:

Certainty of evidence: Bold: Moderate; Italic: Low; Bold and Italic: Very low

Several observational cohort studies that assessed the effects of GLP-1RAs [77–80] and SGLT2i [81–85] on various neuropathic endpoints are not discussed herein due their uncontrolled design constituting a source of substantial bias. It is therefore not surprising that the majority of these observational studies have shown positive results.

Collectively, to date there is no convincing evidence to support favorable, clinically relevant effects of GLP-1RAs and SGLT2i on diabetic neuropathy. However, the certainty of evidence based on published RCTs is generally low. It has been suggested that, apart from a placebo effect on symptoms or signs and measurement noise, the main reason why it is difficult to demonstrate monotonic worsening of neuropathic endpoints appears to be a very slow worsening of DSPN [86, 87]. Given the slow progression of DSPN particularly in the RCT setting, to demonstrate drug efficacy within the relatively short treatments periods in these RCTs, regression of peripheral and autonomic nerve dysfunction rather than mere slowing of progression would be required. Given that a disease modifying pharmacotherapy were able to delay the progression of neuropathy, several years would be required to demonstrate efficacy [86, 88]. Indeed, for example the NATHAN trial demonstrated that more patients achieved a clinically meaningful improvement and fewer showed progression of neuropathic deficits with alpha-lipoic acid than with placebo only after 4 years but not after 2 years of treatment [20].

### Pain disorders

Emerging preclinical and clinical evidence suggests that GLP-1RAs may be effective in pain management. Several preclinical models and clinical studies point to a potential of GLP-1RAs for managing pain through their neuroprotective and metabolic regulatory properties, such as inhibiting inflammation responses and oxidative stress, promoting β-endorphin release, and modulating several other crucial biological pathways [34]. Seven pain modalities, including inflammatory pain, osteoarthritis, visceral pain, neuropathic pain, diabetic neuropathy, cancer pain, and headache have recently been highlighted to support the efficacy and underlying biological mechanisms of GLP-1RAs as therapeutic agents for pain conditions [34].

Osteoarthritis is among the leading causes of disability worldwide, and approximately 4.3% of adults have painful knee osteoarthritis [89]. In the STEP 9 study, 407 non-diabetic participants with obesity (41% with BMI ≥ 40 kg/m^2^) and a clinical and radiologic diagnosis of moderate knee osteoarthritis with at least moderate pain (Western Ontario and McMaster Universities Osteoarthritis Index [WOMAC] mean pain: 71 of 100 points) were randomly assigned, in a 2:1 ratio, to receive once-weekly s.c. semaglutide or placebo. After 68 weeks of treatment, the mean estimated difference for weight loss was -10.5 (95% CI: -12.3 to -8.6)%, while for the change in WOMAC pain score it was -14.1 (95% CI: -20.0 to -8.3) points in favor of semaglutide. Likewise, the percentages of responders with ≥ 50% pain reduction were 65% for semaglutide vs 35% for placebo (number needed to treat: 3.3) [90]. These findings not only suggest that substantial weight loss causes a dramatic reduction in knee pain related to osteoarthritis, but also that appropriately designed longer-term RCTs in patients with diabetes related neuropathic pain using simple questionnaires and clinical tools should be feasible given the high prevalence of this condition.

## Response rates and discontinuations in clinical practice

### Glycemic and weight responses

Substantial interindividual differences in the magnitude of HbA1c reductions and weight loss obtained with GLP-1RAs have been found in both RCTs and clinical practice, which remain poorly understood [1]. Since RCTs often include highly selected, motivated patients who therefore may not be representative of the wide spectrum of individuals with T2D in routine clinical practice, collecting real-world data is necessary to close this gap. Moreover, treatment responses in RCTs may be biased by the generally more intensive care during the course of the trial. Data from 4467 adults with T2D in the Diabetes Patient Follow-up (DPV) registry indicate that only 14% of participants achieved meaningful reductions in both HbA1c (absolute reduction ≥ 5.5 mmol/mol [0.5%]) and body weight (relative reduction ≥ 5%), while 36% achieved an HbA1c only response and 7% experienced a weight-only response after 6 months. These data suggest significant heterogeneity in responses to GLP-1 RAs among individuals with T2D in routine clinical practice [91].

Current evidence does not support the use of biomarkers, clinical phenotypes, or genetic profiling to predict initial glycemic control or weight loss outcomes with GLP-1 therapies. The underlying causes of response variability remain elusive. It has been hypothesized that variations in GLP-1R expression across target tissues, such as the brain or pancreatic islets, may drive these differences [1]. Robust and appropriately powered studies are required to understand treatment effect heterogeneity and evaluate the potential for precision medicine to inform future clinical care [92]. Indeed, recent evidence suggests that a precision medicine approach targeting GLP-1RAs and SGLT2i based on differential glycemic response was associated with a lower risk of developing new-onset microvascular complications [93].

### Discontinuation rates

There is general agreement that treatment of T2D with GLP-1RAs and SGLT2i is long-term if tolerated [22], and the same paradigm applies to pathogenetic pharmacotherapy for diabetic neuropathy [86–88]. Once a SGLT2i is initiated for kidney protection (typically at an eGFR ≥ 20 mL/min/1.73 m^2^), it is reasonable to continue even if the eGFR falls below that threshold. SGLT2i treatment should be discontinued only if it is no longer tolerated or if the patient progresses to dialysis or kidney transplantation [22]. The recent WHO guideline recommends that in adults living with obesity, GLP-1RAs may be used as a long-term treatment for obesity (conditional recommendation, moderate certainty evidence) [94]. The extent and temporal sequence of weight regain after cessation of GLP-1RA treatment have been estimated in a recent meta-analysis. The monthly rate of weight regain was 0.6 (95% CI: 0.4–0.8) kg for GLP-1RAs and 0.8 (0.6–0.9) kg for the newer and more effective GLP-1RAs. The time to no difference between intervention and control was 1.1 (0.6–1.5) years for GLP-1RAs and 1.3 years (0.8–1.8) for the newer and more effective GLP-1RAs after cessation. Weight regain was faster after cessation of weight management medications compared to behavioral weight management programs (by 0.3 kg [0.22–0.34] monthly) [95].

In RCTs of GLP-1RAs and SGLT2i the proportions of participants discontinuing treatment ranged widely from 10–40% [96]. RCTs are conducted under strictly controlled conditions where patients are closely monitored and encouraged to adhere to their treatment. Consequently, discontinuation rates are typically expected to be higher in real-world clinical practice. Conversely, unlike trial participants, patients in routine care have the flexibility to switch between medications within the same class, a factor that may actually reduce treatment discontinuation rates.

Discontinuation rates for GLP-1RAs and SGLT2i after 1 year were approximately 20%, while after 3 years these rates were 32% and 40% in Denmark [97–99]. In a Swedish study, participants were considered to be on treatment if refills occurred within the calculated supply period plus a 90-day grace period, while exceeding this interval was defined as treatment discontinuation. These authors found that while approximately 50% of T2D patients discontinued GLP-1RA or SGLT2i treatment within five years, more than half of them eventually restarted. Thus, the actual proportion of patients on active treatment remained between 70–80% throughout the five-year follow-up period [96]. In the population-based Scottish Care Information Diabetes Collaboration database, the 1-year discontinuation rates in total among individuals with T2D were 25.9% for dapagliflozin, 24% for empagliflozin, 21–22% for dulaglutide, liraglutide and semaglutide, and 36% for lixisenatide, 30% for exenatide SR, and 29% for exenatide, of which 5.5–9% were due to intolerance [100].

In a 5-month US study including commercially insured adults who started semaglutide s.c. for weight management, most users deviated from the recommended monthly dose-escalation schedule and 46% had discontinued the treatment. Discontinuation was strongly associated with copayment amount and lower household income and education level, pointing to financial barriers as important challenges in the delivery of evidence-based care in real-world settings [101]. In GLP-1RA users with T2D or obesity in the US who were prescribed dulaglutide, exenatide, liraglutide, or semaglutide, the discontinuation rate was 37% at 1 year [102]. Another US retrospective cohort study including adults with overweight or obesity without T2D who initiated injectable semaglutide or tirzepatide reported that 81% had low maintenance dosages. During the first year, 22% of patients who initiated treatment with semaglutide and 16% of those with tirzepatide discontinued obesity pharmacotherapy within 3 months, while 31% with semaglutide and 34% with tirzepatide discontinued within 3–12 months. The average weight reduction in this cohort was lower than that observed in the phase 3 trials, likely because of higher rates of discontinuation and lower maintenance dosages [103]. Overall, 48% discontinued their medication due to cost or insurance-related issues, 15% due to inability to tolerate the side effects, and 12% because they were unable to fill the medication due to shortages [104].

These data point to lower glycemic and weight responses to GLP-1RA or SGLT2i treatments in real-world scenarios when compared to RCTs. Higher discontinuation rates of GLP-1RA medications in real-world settings when compared to RCTs were primarily due to high cost or insurance-related barriers (US), but adverse events and medication shortages were also relatively common causes.

## Risks in real-world practice

### GLP-1RA specific risks

While GLP-1RAs have demonstrated significant therapeutic benefits, their clinical use is frequently complicated by a distinct profile of adverse events that impact both patient safety and long-term tolerability. Careful consideration of potential benefits and risks is essential for optimizing treatment outcomes and ensuring patient safety when using GLP-1RAs in clinical practice. The pharmacological slowing of gastric emptying associated with GLP-1RAs increases the likelihood of retained gastric contents which potentially could contribute to an increased risk for pulmonary aspiration during procedures involving upper gastrointestinal endoscopy or general anesthesia [105]. Significant weight loss frequently entails a reduction in lean body mass, potentially predisposing individuals to sarcopenic phenotypes. Furthermore, patients may experience sight-threatening ocular complications presumably linked to the rapid improvement of glycemic control. Although early safety concerns regarding pancreatic cancer have been largely resolved by data from long-term clinical trials, emerging evidence suggests a potential, albeit small, increased risk for thyroid malignancies that warrants ongoing surveillance [105].

### Gastrointestinal adverse events

Reports of GLP-1 RA exposures increased substantially after the 2021 approval for weight-loss indications, with semaglutide accounting for most of the post-approval growth. Although the majority of exposures produced mild and self-limited gastrointestinal symptoms, the period was also marked by a significant rise in healthcare facility referrals, increasing from 23% before approval to 34% after approval. This pattern indicates that even clinically non-severe exposures can generate considerable healthcare utilization [106]. The rising use of GLP-1RAs brings concerns about side effects, long-term outcomes, and unregulated products. Ensuring safe access requires oversight, monitoring, and coordinated clinical care [107].

A post-marketing pharmacovigilance analysis using the FDA Adverse Event Reporting System (FAERS) for tirzepatide revealed several new signals including postmenopausal haemorrhage and menstrual disorder (implying regulatory interference on sex hormones), Wernicke’s encephalopathy and sleep disorder (malnutrition due to low intake). Pancreatitis, impaired gastric emptying, dehydration and cholelithiasis carried higher risks with serious clinical outcomes. Sleep disorder, delayed gastric emptying, and medullary thyroid cancer were more common in men, while starvation ketoacidosis and incorrect injection site were more common in women [108]. The UK MHRA recently issued a new warning after a rise in reported deaths from pancreatitis in GLP-1RA users [109].

### Adverse consequences of delayed gastric emptying

Delayed gastric emptying is a hallmark of diabetic gastroparesis due to autonomic neuropathy. Since GLP-1RA treatment may slow gastric emptying, the question arises whether it is associated with an increased incidence of gastroparesis. Indeed, a US retrospective cohort study found an increased incidence of gastroparesis among individuals with obesity without T2D who were using semaglutide as compared with bupropion-naltrexone (adjusted HR: 3.33 (2.27–4.98) and sleeve gastrectomy (adjusted HR 6.14 (3.94–9.57) [110]. In another US database the use of GLP-1RAs compared with bupropion-naltrexone was also associated with an increased risk of gastroparesis (HR: 3.67 (1.15–11.90) [111].

A recent meta-analysis quantified the risk of delayed gastric emptying associated with GLP-1RA treatment assessed by scintigraphy or a stable isotope breath test. GLP-1RA therapy increased gastric emptying half-time by a standardized mean difference (SMD) of 2.38 (95% CI: 1.05–3.71), corresponding to a mean prolongation of 74 (95% CI: 46–101) min. Furthermore, a trend was observed towards a more pronounced effect with short-acting GLP-1RAs (SMD: 3.86 (2.37–5.35) corresponding to a prolongation of 116 (71–161) and early treatment phases (< 10 weeks) (SMD: 2.72 (1.15–4.35) with a mean prolongation of 82 (35–131) [112].

Several meta-analyses assessed the risk of retained gastric contents causing premature termination of endoscopy and pulmonary aspiration in individuals treated with GLP-1RAs [113–117] (Table 7). All analyses found an increased risk of retained gastric contents and premature termination of endoscopy in GLP-1RA users compared to the control groups. Except for the study by Singh et al. [113], the ORs ranged from 4.54 to 6.30 for the risk of retained gastric contents and from 2.19 to 5.13 for aborted procedures. Singh et al. [113] reported considerably higher ORs, but accordingly wide 95% CI ranges. All analyses did not find an increased risk of pulmonary aspiration in GLP-1RA users compared to the controls (Table 7). In the analysis by Tarar et al. [116] individuals taking tirzepatide had the highest rate (18.9%) of retained gastric residue, followed by semaglutide (15.6%), dulaglutide (10.9%), liraglutide (8.1%), and exenatide (6.2%). Thus, GLP-1RA treatment is clearly associated with a substantial risk of retained gastric contents and aborted procedures, with presumably the highest risk observed for tirzepatide and semaglutide among the individual GLP-1RA compounds. However, this hazard does not seem to contribute to an increased risk of pulmonary aspiration.

**Table 7:** Results of meta-analyses assessing the risk of retained gastric contents causing premature termination of endoscopy and pulmonary aspiration

**Author**	**n** **(GLP-1RA/ control)**	**Retained gastric contents** **OR (95% CI)**	**Aborted procedures** **OR (95% CI)**	**Pulmonary aspiration** **OR (95% CI)**
Singh et al. [113]	4,449/72,703	15.39 (4.65–50.99)	13.86 (4.42–43.43)	21.06 (0.13–3379)
Facciorusso et al. [114]	19,842/60,035	5.56 (3.35–9.23)	5.13 (3.01–8.75)	4.04 (0.63–26.03)
Baig et al. [115]	96,521/165.497	4.54 (3.30–6.24)	4.54 (3.05–6.75)	0.96 (0.53–1.75)
Tarar et al. [116]	32,144/73,273	6.30 (5.30–7.49)	2.19 (1.42–3.38)	1.26 (0.86–1.87)
Elkin et al. [117]	73,984/111,430	5.96 (3.96–8.98)	n.a	1.04 (0.87–1.25)

n.a.: not available.

Clinical recommendations regarding the preoperative management of GLP-1RAs are inconsistent. Guidelines from the American Association of Nurse Anesthesiology (AANA) [118] and an American multisociety clinical practice guidance [119] suggested considering holding daily GLP-1RA formulations on the day of the procedure/surgery, while withholding weekly formulations a week prior, particularly when an elevated risk of delayed gastric emptying and aspiration exists. Conversely, a multidisciplinary consensus statement endorsed by various British clinical societies [120, 121] advised against routine cessation, suggesting that patients maintain their medication throughout the perioperative period. These societies argue that brief pauses are unlikely to reverse delayed gastric emptying due to the drugs’ long-acting nature and may instead lead to poor glycemic control. Nonetheless, the Medicines and Healthcare products Regulatory Agency (MHRA) advised that healthcare professionals should be aware of the potential risk of pulmonary aspiration in patients using GLP-1 or dual GIP/GLP-1RAs who undergo surgery or procedures with general anaesthesia or deep sedation [120]. Despite global concerns regarding the perioperative safety of GLP-1RAs, high-quality clinical evidence remains scarce. Discrepancies between major guidelines complicate clinical decision-making, underscoring an urgent need for robust, well-powered, specifically randomized controlled or large-scale pragmatic trials. Given the surging popularity of GLP-1RAs, rapid intervention is essential to establish safe management protocols and minimize preventable drug-related morbidity [122].

### Reduced skeletal muscle mass

Since GLP-1RA treatment may be associated with profound weight loss, concerns have emerged for potential adverse effects on muscle quantity, composition, and function [123]. Weight reduction is often accompanied by a decline in fat-free mass (FFM), prompting concerns as to the development of sarcopenia. Specifically, FFM accounted for 39% of total weight loss with semaglutide compared to 25% with tirzepatide. It should be noted, however, that utilizing FFM as a proxy for muscle mass has inherent limitations, as few studies provide direct measurements of skeletal muscle [105]. It has also been argued that skeletal muscle changes with GLP-1RA treatments appear to be adaptive rather than maladaptive, since changes in muscle volume z-scores reflect shifts consistent with the expected outcomes of aging, disease progression, and achieved weight loss. [123]. Furthermore, improvements in insulin sensitivity and muscle fat infiltration induced by GLP-1RAs likely facilitate an adaptive process that enhances muscle quality, thereby reducing the risk of declining strength and physical function [123].

While a primary objective of contemporary pharmacological obesity management is to maximize fat reduction while maintaining lean muscle mass and functionality, the available evidence is inconclusive regarding the impact of GLP-1-based therapies on muscle health. It has been emphasized that future studies should assess not only skeletal muscle mass, but also muscle architecture, strength, and physical performance, particularly in populations vulnerable to sarcopenia, such as the elderly and frail [124]. Of note, the development of compounds to maintain or improve muscle mass designed in combination with GLP-1RAs is currently underway [123].

### Increased resting heart rate

Resting heart rate is a fundamental determinant of cardiac output and physiological homeostasis. Despite its simplicity, it serves as a critical prognostic marker for adverse outcomes and the onset of disease in healthy populations. Although heart rate integrates sympathetic and parasympathetic signaling with intrinsic sinus rhythm, the mechanisms linking it to long-term health remain obscure, particularly in otherwise healthy individuals. Furthermore, while targeted heart rate reduction may be therapeutic in specific disorders, the underlying reason why it influences outcomes in the general population is yet to be fully elucidated [125]. An elevated resting heart rate is a predictor of cardiovascular morbidity and mortality in individuals with and without diabetes [126, 127]. In patients with heart failure with sinus rhythm, the heart rate threshold beyond which an increase in mortality has been observed is 70 bpm [128], while an increase in heart rate by 5 bpm was associated with a higher risk of all-cause mortality and the composite endpoint of cardiovascular death or hospitalization for heart failure (adjusted HR: 1.06, 95% CI: 1.05–1.08) [129]. As a sign, albeit not specific, of decreasing parasympathetic modulation and increasing sympathetic tone, resting heart rate is increased in people with diabetes and CAN [130, 131].

GLP-1 is known to induce changes in haemodynamic variables and modulate autonomic activity. Exendin has been shown to increase heart rate and diminish vagal activity in mice, while peripheral or central GLP-1, administration has been shown to increase sympathetic activity in rats [126]. A recent meta-analysis confirmed the association of GLP-1RAs increased resting heart rate in overweight or obese patients without diabetes [132] (Table 8). The mean increase compared to controls was highest for orforglipron (7.3 bpm), followed by oral semaglutide (4.5 bpm) and retatrutide (3.5 bpm), a triple GLP-1, GIP, and glucagon receptor agonist. The increase in heart rate compared to control for GLP1RAs in total was 3.5 bpm (Table 8). Network meta-analysis showed that orforglipron 36 mg was associated with the most prominent increase in heart rate (9.29 [4.45–13.86]), whereas tirzepatide 5 mg was not associated with an increase (0.52 [-2.71–3.78]) [129]. In the ACHIEVE-3 trial, the mean increase in heart rate over 1 year was greater with orforglipron (12 mg: 3.7 bpm; 36 mg: 4.7 bpm) than with oral semaglutide (7 mg: 1.0 bpm; 14 mg: 1.5 bpm) [133]. Overall, these data suggest that the risk of increased heart rate mediated by GLP-1RAs should not be underestimated, particularly in individuals with CAN. Possible mechanisms for GLP-1RA-induced heart rate increase include sympathetic activation resulting from higher endogenous insulin release or direct effects on cardiomyocyte receptors [48]. However, a meta-analysis of six studies found a rise in heart rate by 3.45 bpm, but no change in sympathovagal balance (LF/HF ratio) [134].

**Table 8:** Association of GLP-1 receptor agonists with increased heart rate in overweight or obese patients without diabetes in a meta-analysis of 12 studies (adapted from ref. 132)

**Compound**	**n (trials)**	**n (active/control)**	**Mean difference** **(bpm)**	**95% CI**
Liraglutide	4	4085/2097	2.37	1.86–2.89
Semaglutide (s.c.)	4	1991/1096	3.35	1.69–5.01
Semaglutide (oral)	1	334/333	4.50	3.11–5.89
Tirzepatide	2	2231/2264	2.05	0.96–3.13
Retatrutide	1	267/280	3.46	1.74–5.18
Orforglipron	1	220/200	7.30	5.48–9.12
***GLP-1 RAs total***	12	9128/6270	3.47	2.65–4.29

### Asymmetric neuropathies

Lumbosacral radiculoplexus neuropathy (LRPN) and common fibular neuropathy (CFN) are known to be associated with weight loss, while the onset of diabetic LRPN (DLRPN) has been linked to rapid improvement of glycemic control [135]. A retrospective case-controlled study identified 26 individuals who developed 27 DLRPN episodes, with median symptom onset 6 (range: 3–35) months after GLP-1RA initiation, who experienced a 14% weight loss and 2.4% HbA1c reduction. Among 77 individuals with CFN, 82 episodes developed, with a mean GLP-1RA duration of 15 (range: 1–112) months and consecutive weight loss of 16% and HbA1c reduction of 1.2%. Compared with controls, GLP-1RA users were 51% more likely to develop DLRPN (OR 1.5, 95% CI 1.2–1.9) and 30% more likely to develop CFN (OR 1.3, 95% CI 1.0–1.5). The frequency of these conditions has likely increased in parallel with rising GLP-1RA prescriptions. It has been suggested that clinicians should monitor for these neuropathies in patients undergoing rapid glycemic control or significant weight loss [135].

Another series including six patients with DLRPN and five cases with mononeuropathies associated with rapid weight and changes due to GLP-1 RA treatment have recently been reported [136, 137].

### Nutritional deficiencies

GLP-1RA therapy may increase the risk of micronutrient deficiencies due to suppressed appetite, slower gastric emptying, and changes in nutrient absorption. A recent meta-analysis identified vitamin D deficiency as the most prevalent issue, affecting 13.6% of users after one year. Notably, ferritin levels were 26–30% lower with GLP-1RAs than SGLT2i. With the majority of users failing to meet dietary targets for protein, calcium, and iron, these findings highlight a link between GLP-1RA use and significant nutritional gaps. However, as these results originate largely from observational data, a direct causal link remains to be proven. Nonetheless, targeted nutritional assessments may be considered in high-risk patients [138].

Even when the absolute rates for B vitamin deficiencies are modest [139], structured screening and prevention are recommended by experts as core priority of care to mitigate nutritional risks and clinical consequences [140, 141]. In contrast, short‑term SGLT2i data do not indicate thiamine depletion [142].

### Thyroid cancer

Since most human medullary thyroid carcinomas and hyperplastic C cells express GLP-1 receptors, a personal or family history of medullary thyroid carcinoma or multiple endocrine neoplasia type 2 (MEN2) is contraindicated for the use of GLP-1RAs [105]. A French health insurance study reported that the use of GLP-1RAs for 1–3 years was associated with an increased risk of all thyroid cancer (HR 1.58, 95% CI 1.27–1.95) and medullary thyroid cancer (HR 1.78, 95% CI 1.04–3.05) [143]. A recent meta-analysis of CVOTs found 6 vs 2 cases of spontaneous medullary thyroid carcinoma with GLP-1RA treatment vs placebo, respectively [144].

### Non-arteritic anterior ischemic optic neuropathy (NAION)

NAION represents an infrequent yet serious cause of adult vision loss. While its exact pathophysiology remains elusive, the condition is widely attributed to hypoperfusion of the optic nerve head, which triggers localized edema and subsequent fiber infarction [102]. In a meta-analysis of 15 longitudinal studies (n > 1.5 million patients) GLP-1RA use was associated with higher risk of NAION (OR 1.70; 95% CI 1.23–2.36), consistent across randomized (OR 2.36; 0.85–6.53) and non-randomized studies (OR 1.64; 1.15–2.35). Absolute NAION risk in the GLP-1RA group was 0.09%, corresponding to a 0.04% risk difference with a number needed to harm (NNH) ∼2,700. There was no association with overall ocular events (OR 0.95; 95% CI 0.86–1.05) [145].

In an active-comparator, new-user, target trial emulation analyzing a nationwide cohort of US veterans with T2D followed over up to 7.5 years, semaglutide initiators showed an increased HR for NAION at 2.33 (95%CI, 1.54–3.54). The incidence rate of NAION was 123/100,000 person-years among semaglutide users and 67/100 000 person-years among SGLT2i users [146]. Likeweise, the majority of the numerous cohort studies reported an increased risk of NAION in GLP-1RA users. In the U.K. Clinical Practice Research Datalink cohort GLP-1RAs were associated with an increased risk of NAION compared with DPP-4 inhibitors (RR 2.56; 95% CI 1.44–4.86) after 1 year in adults with T2D, particularly in younger individuals, men, ever-smokers, and those with a marked hemoglobin HbA1c reduction [147]. A retrospective cohort analysis from the TriNetX Global Collaborative Network also demonstrated an increased risk of NAION (risk difference 0.022%, 95% CI 0.01%-0.034%; risk ratio 1.3439, 95% CI 1.14–1.58) [148]. In contrast, in a recent meta-analysis of 20 RCTs including 83,288 participants with T2D or cardiometabolic diseases, GLP-1RAs were not associated with an increased risk of optic nerve and vision-threatening severe AEs, including ischemic optic neuropathy, blindness, papilledema, blurred vision, visual impairment, or reduced visual acuity [149].

The mechanisms linking GLP-1RAs to NAION remain unclear, but may include hypotension, rapid glycemic improvement with transient microvascular dysregulation, and impaired vascular autoregulation at the optic nerve head [146]. In summary, the majority of studies point to a modestly increased risk of NAION due to GLP-1 RA treatment. Although the absolute risk is low, clinicians and patients alike should be aware the rare but evident risk.

### SGLT2i specific risks

In a meta-analysis of the CVOTs in participants with T2D, SGLT2i treatment was associated with an increased risk of volume depletion (incident rate ratio (IRR) 1.17 [95% CI: 1.02–1.33]), genital infection (IRR 3.50 [95% CI: 3.09–3.95]), urinary-tract infections compared with control treatment (IRR 1.10 [95% CI 1.02,1.18]), amputation ( [IRR] 1.20 [95% CI: 1.05–1.38]), and diabetic ketoacidosis (IRR 2.41 [95% CI: 1.63–3.57]) compared to control treatment [150]. Another meta-analysis assessed the risk and incidence of serious AEs associated with SGLT2 treatment compared to placebo in participants with T2D [25]. The following hazard ratios (HR [95% CI]) and incidence rates were reported for SGLT2i vs placebo: bone fracture: 1.09 (1.00–1.19) with 18 and 17 events/1000 patient-years, lower limb amputation: 1.16 (1.01–1.34) with 7.6 and 6.9 events/1000 patient-years, serious urinary tract infection (death, life-threatening, hospitalization, or other important medical event in the opinion of an investigator): 1.00 (0.85–1.16) with 6.8 and 6.1 events/1000 patient-years, and ketoacidosis: 2.29 (1.42–3.71) with 0.9 and 0.6 events/1000 patient-years. Thus, serious AEs were relatively rare, but significantly higher for SGLT2i for bone fracture, lower limb amputation, and ketoacidosis [25].

## Unresolved issues

### Diabetic retinopathy

In a meta-analysis including 61 RCTs GLP1-RAs (OR 1.08; CI 95% 0.94–1.23) and SGLT2i (OR 1.02; CI 95% 0.76–1.37) were not associated with an increased risk of diabetic retinopathy (DR) in participants with T2D. Notably, empagliflozin was associated with a lower risk of DR, but that sub-analysis included only three RCTs (OR 0.38; 95% CI 0.17–0.88) [151]. However, some cohort studies point to an increased risk of DR in GLP-1 users. In a retrospective cohort analysis, tirzepatide use was significantly associated with new-onset proliferative DR (OR 2.15 [95% CI 1.24–3.74]). On the other hand, tirzepatide was also associated with reduced incidence of DR (OR 0.73 [95% CI 0.62–0.86]) [152]. An analysis of the TriNetX database showed that the use of GLP-1 RAs was associated with an increased incidence of DR (HR 1.07; 95% CI, 1.03–1.11). In a subgroup analysis of individuals with preexisting DR, GLP-1 RAs were not associated with progression to proliferative DR (HR 1.06; 95% CI, 0.97–1.15) or diabetic macular edema (HR, 0.98; 95% CI, 0.95–1.01), but were associated with a lower occurrence of vitreous hemorrhages (HR 0.74; 95% CI, 0.68–0.80), neovascular glaucoma (HR, 0.78; 95%CI, 0.68–0.88), or blindness (HR, 0.77; 95% CI, 0.73–0.82) [153]. In a recent analysis from Taiwan’s National Health Insurance Research Database including > 1 million individuals with T2D without macrovascular or microvascular diseases at initiation, SGLT2i users showed a lower risk of vision-threatening retinopathy (HR 0.78 [95% CI: 0.62–0.98]) and amputation (HR 0.61 [0.46–0.89]) than GLP-1RA users [154].

Collectively, the available evidence on the effects of GLP-1RAs is inconsistent. Further studies are needed to decipher the effects of GLP-1RAs on DR. Indeed, the 5-year FOCUS RCT focusing on the effects of semaglutide on retinopathy progression and complications is currently underway to further elucidate the risks and benefits associated with semaglutide treatment in T2D (ClinicalTrials.gov NCT03811561; reporting in 2027).

### Diabetic foot disease

In a recent meta-analysis GLP-1RA use was not associated with an increase in revascularization (OR: 0.87 (95% CI 0.73–1.05) or amputation outcomes (OR: 0.82 (0.53–1.27) in individuals with diabetes and peripheral arterial disease (PAD) [155]. In a retrospective cohort analysis of individuals with incident diabetic foot ulcers from the French National Health Data System, protective factors included GLP-1RA use, lipid-lowering therapy, and structured, multidisciplinary follow-up. Similar patterns were observed for post-amputation mortality, with GLP-1RA use showing a consistent survival benefit [156].

Several cohort studies compared the use of GLP-1RAs and SGLT2i focusing on diabetic foot disease. In a recent analysis of the nationwide U.S. electronic health records database, GLP-1RA use in individuals with T2D and PAD was associated with lower risks of major amputation (HR 0.79 [95% CI 0.70–0.90]) and revascularisations (HR 0.82 [0.76–0.88]) compared to SGLT2i treatment, with semaglutide and tirzepatide showing the greatest benefit [157]. Likewise, a TriNetX analysis showed that GLP-1RA treatment was associated with greater major amputation-free survival compared with SGLT2i in people with T2D in general and in a subgroup with PAD [158]. These data support a potential vascular benefit by GLP-1RAs that warrants prospective evaluation.

McCoy et al. [159] emulated a target trial using two linked US national claims databases. They observed greater relative risk of lower extremity complications with sulfonylurea use compared to GLP-1RA and SGLT2i use, and with SGLT2i use compared to GLP-1RA use (HR 1.11; 95% CI 1.03–1.21). However, it should be considered that the absolute differences between the medication classes were small (< 1%). In contrast, in another emulated target trial analyzing a 6-year follow-up of the Danish national health care registry cohort, any foot disease occurred in 10.8% of SGLT-2i users and 12.0% of GLP-1RA users (RR 0.90 (95% CI: 0.84–0.97. This modest reduction in risk among SGLT-2i users was driven by lower risk for neuropathy (RR 0.78 [0.68–0.87]). Users of SGLT-2is and GLP-1RAs had similar risks for peripheral artery disease, foot ulcers, amputations, and all-cause mortality [99].

Collectively, these real-world studies point to slightly better outcomes for diabetic foot disease in GLP-1RA rather than SGLT2 user, but the absolute differences between groups were small, and it remains unclear whether they reflect a causal effect.

### Possible complementary strategies in DSPN due to distinct mechanistic pathways

Pathogenetic pharmacotherapies for DSPN engage intracellular glucose‑toxicity pathways in nerve tissue. For example, α‑lipoic acid exerts antioxidant and anti-inflammatory properties, including reactive oxygen species scavenging, metal chelation, and the regeneration of endogenous antioxidants such as glutathione [160]. Furthermore, benfotiamine upregulates transketolase, diverting excess glucose away from polyol, hexosamine, protein kinase C, and advanced glycation end product pathways [161]. These mechanisms address residual cellular injury that may persist despite improved glycemia, which is mechanistically distinct from the upstream metabolic unloading mediated by GLP‑1RAs or SGLT2i. In the clinical setting the highly favorable safety profile of these compounds qualifies them particularly for long-term treatment [7].

Although emerging evidence indicates that GLP‑1RA and SGLT2i may also lower systemic oxidative stress and inflammation [162–164], their actions do not replicate the specific effects attributed to the aforementioned compounds within peripheral nerves. Moreover, in absence of evidence for the efficacy of GLP‑1RAs and SGLT2i from appropriate RCTs in diabetic neuropathy, the established multimodal DSPN management approach (i.e. causal, pathogenetic, and symptomatic treatment) remains pertinent in the current therapeutic landscape. Since novel antidiabetic agents can lessen upstream substrate and oxidative load, while pathogenetic drugs counter residual intracellular damage, a complementary strategy seems reasonable that could be particularly relevant for high-risk groups or individuals who do not respond adequately despite modern glucose‑lowering therapy, including those for whom GLP‑1RA–related micronutrient risks warrant monitoring (e.g. B vitamins).

## Conclusion and outlook

There is an unmet need for novel pharmacotherapies derived from the pathogenetic concepts of diabetic neuropathy. Over the past decade, the management of T2D has been transformed by GLP-1RAs and SGLT2i, both of which offer therapeutic advantages extending far beyond blood glucose regulation. Despite a sustainable preclinical basis, there is a lack of evidence from RCTs to support favorable effects of GLP-1RAs and SGLT2i on neuropathy outcomes largely due to poor study designs with small, inadequately defined study samples, short duration, or questionable outcomes. Unfortunately, previous large-scale long-term CVOTs did not take the opportunity to include some simple tools to assess neuropathy and thereby to provide some relevant insights. Several possible risks associated with GLP-1RAs and SGLT2i treatment in real-world practice have been identified, but worldwide efforts to establish safe management protocols to minimize preventable drug-related morbidity should be harmonized. In the future, well designed RCTs of adequate duration and sample size are required to determine the efficacy of GLP-1RAs and SGLT2i on clinical endpoints including neuropathic deficits, pain, and patient-reported outcomes in individuals with diabetic neuropathy. In addition, neuropathies linked to prediabetes, obesity, and metabolic syndrome should become a therapeutic target of these drug classes. Only in this way will it be possible to exploit the full therapeutic potential of these widely used pharmacotherapies.

## Data Availability

No datasets were generated or analysed during the current study.
